# The dipeptidyl peptidase-4 (DPP-4) inhibitor teneligliptin functions as antioxidant on human endothelial cells exposed to chronic hyperglycemia and metabolic high-glucose memory

**DOI:** 10.1007/s12020-016-1052-0

**Published:** 2016-08-16

**Authors:** Gemma Pujadas, Valeria De Nigris, Francesco Prattichizzo, Lucia La Sala, Roberto Testa, Antonio Ceriello

**Affiliations:** 1 Institut d’Investigacions Biomèdiques August Pi i Sunyer (IDIBAPS) and Centro de Investigación Biomédica en Red de Diabetes y Enfermedades Metabólicas Asociadas (CIBERDEM), Hospital Clínic, C/Rosselló, 149-153, 08036, Barcelona, Spain; 2 Department of Clinical and Molecular Sciences, DISCLIMO, Università Politecnica delle Marche, Ancona, Italy; 3 Experimental Models in Clinical Pathology, INRCA-IRCCS National Institute, Ancona, Italy; 4 IRCCS MultiMedica, Milan, Italy

**Keywords:** Diabetes, High glucose, Metabolic memory, Endothelial dysfunction, Antioxidant, DPP-4 inhibitors, Teneligliptin and sitagliptin

## Abstract

Dipeptidyl peptidase-4 inhibitors are widely used in type 2 diabetes. Endothelium plays a crucial role maintaining vascular integrity and function. Chronic exposure to high glucose drives to endothelial dysfunction generating oxidative stress. Teneligliptin is a novel dipeptidyl peptidase-4 inhibitor with antioxidant properties. This study is aimed to verify a potential protective action of teneligliptin in endothelial cells exposed to high glucose. Human umbilical vein endothelial cells were cultured under normal (5 mmol/L) or high glucose (25 mmol/L) during 21 days, or at high glucose during 14 days followed by 7 days at normal glucose, to reproduce the high-metabolic memory state. During this period, different concentrations of teneligliptin (0.1, 1.0 and 3.0 µmol/L) or sitagliptin (0.5 µmol/L) were added to cells. Ribonucleic acid and protein expression were assessed for antioxidant response, proliferation, apoptosis and endoplasmic reticulum stress markers. Teneligliptin promotes the antioxidant response in human umbilical vein endothelial cells, reducing ROS levels and inducing Nrf2-target genes messenger ribonucleic acid expression. Teneligliptin, but not sitagliptin, reduces the expression of the nicotine amide adenine dinucleotide phosphate oxidase regulatory subunit *P22* ^*−phox*^, however, both blunt the high glucose-induced increase of *TXNIP*. Teneligliptin improves proliferation rates in human umbilical vein endothelial cells exposed to high glucose, regulating the expression of cell-cycle inhibitors markers (*P53*, *P21* and *P27)*, and reducing proapoptotic genes (*BAX* and *CASP3)*, while promotes *BCL2* expression. Teneligliptin ameliorates high glucose-induced endoplasmic reticulum stress reducing the expression of several markers (*BIP, PERK, ATF4, CHOP, IRE1a* and *ATF6)*. Teneligliptin has antioxidant properties, ameliorates oxidative stress and apoptotic phenotype and it can overcome the metabolic memory effect, induced by chronic exposure to high glucose in human endothelial cells.

## Introduction

Type 2 diabetes mellitus (T2DM) patients have a doubled risk to suffer from cardiovascular diseases (CVD) compared to non-diabetic individuals after adjustment for other cardiovascular risk factors [1]. Substantial clinical and experimental evidences suggest that endothelial dysfunction (ED) is a crucial early step in the development of CVD [2]. Continuous exposure to high-glucose levels is accepted as the major factor implicated in the pathogenesis of diabetic vascular complications [3]. Several lines of evidence indicate that hyperglycemia causes tissue damage mostly through reactive oxygen species (ROS) overproduction [3, 4], increasing oxidative stress and fostering the development of ED [3]. However, lowering glucose levels is not sufficient to switch off this intracellular pro-oxidant environment [5], a pathogenic mechanism partly explaining the “metabolic memory” concept, defined like the perpetuation of vascular damage despite the achievement of improved glycemic control [6]. In vitro experiments on cultured endothelial cells (ECs), as well as in vivo studies on diabetic animals, show a ROS-mediated persistence of vascular stress after glucose normalization, with long standing markers of oxidative stress and overactivation of pathways widely linked to the pathogenesis of ED and diabetic complications [5].

Glucagon-like peptide 1 (GLP-1) improves endothelial function in diabetes [7–9], so therapeutic strategies for patients with T2DM are focused in increasing the incretin response, either by inhibiting dipeptidyl peptidase-4 (DPP-4) activity or by using degradation-resistant GLP-1 analogues [7]. DPP-4 is an ubiquitously expressed transmembrane glycoprotein that cleaves N-terminal dipeptides from a variety of substrates [7], including the incretin hormones GLP-1 and gastric inhibitory polypeptide (GIP).

Among DPP-4i, it is recent the commercialization of the potent, long-lasting DPP-4i, teneligliptin. It has a unique structure characterized by five consecutive rings, explaining its powerful activity. Teneligliptin has a half-life of 24.2 h, with resulting DPP-4 inhibition throughout the day, and is eliminated by hepatic metabolism or excreted from the kidney as the unchanged drug, meaning that a dose adjustment is not required, even for patients with renal or hepatic disorders [10]. The efficacy and safety of long-term use of teneligliptin in monotherapy or combination therapy were evaluated in T2DM patients [11]. DPP-4i have shown an antioxidant capacity [12], however, no studies have evaluated a possible difference between them, as well as if this property can impact on HG-induced damage to ECs.

The present study was aimed to explore the effects of the DPP-4i teneligliptin, compared to sitagliptin, on ED and antioxidant response in ECs exposed to high-glucose conditions.

## Materials and methods

### Cell culture and experimental design

Human umbilical vein endothelial primary cells (HUVECs) were purchased by Lonza and cultured with EGM™-2 Bulletkit™ (LonzaIbérica S.A.U., Barcelona, Spain) adding supplemental growth factors: epidermal growth factor, hydrocortisone, human recombinant fibroblast growth factor-beta, heparin, 2 % fetal bovine serum and gentamicin/amphotericin-B, provided with the media, at 37 °C in a humidified atmosphere with 5 % CO_2_. Cells were used between 4–6 passages. HUVECs were seeded and allowed to attach overnight. Next day cells were exposed to one of three glucose experimental conditions with or without teneligliptin (at 0.1, 1.0 or 3.0 µmol/L) or sitagliptin (at 0.5 µmol/L) [13]: continuous normal glucose (NG-5 mmol/L) for 21 days; continuous high glucose (HG-25 mmol/L) for 21 days; and high-metabolic memory (HM-continuous HG for 14 days, followed by NG for the last 7 days) [6, 14]. HUVECs were cultured during the 3 weeks changing the media each 48 h and without passaging the cells.

Teneligliptin hydrogen bromide hydrate (3-[(2S,4S)-4- [4-(3-methyl-1-phenyl-1H-pyrazol-5-yl)piperazin-1-yl]pyrrolidin-2-yl-carbonyl]thiazolidinehemipentahydrogenbromide hydrate) was kindly provided by Mitsubishi Tanabe Pharma Corporation (Osaka, Japan).Sitagliptin phosphate monohydrate (7-[(3R)-3-amino-1-oxo-4-(2,4,5-trifluorophenyl)butyl]-5,6,7,8-tetrahydro-3-(trifluoromethyl)-1,2,4-triazolo[4,3-a]pyrazine phosphate) was provided by BioVision (San Francisco, USA).

### RNA isolation and real-time polymerase chain reaction (qRT-PCR)

Total RNA was isolated from HUVECs using Total RNA isolation kit (NorgenBiotek Corp, Thorold, Ontario, Canada) following the manufacturer’s instructions. First-strand complementary deoxyribonucleic acid (DNA) was prepared using 1–2 µg of total RNA, the Superscript III RT kit and random hexamer primers (Invitrogen, Carlsbad, CA, USA) in a total volume of 25 µl according to the manufacturer’s instructions. Reverse transcription reaction was carried for 90 min at 50 °C and an additional 10 min at 55 °C. Real-time PCR (qRT-PCR) was performed on an ABI Prism 7900 sequence detection system using Sybr Green reagents (Takara Bio Company, Clontech, Mountain View, CA, USA) and TaqMan® Gene Expression Master Mix (Life Technologies, Madrid, Spain).

### Protein analysis

For western blot analysis HUVECs were lysed in Radioimmunoprecipitation assay buffer (Sigma-Aldrich) adding 10 % proteases and 1 % phosphatases inhibitors (Sigma-Aldrich Química, S.L., Madrid, Spain). Protein content was determined using Bradford assay buffer (Sigma-Aldrich Química, S.L., Madrid, Spain). Fifty micrograms of lysates were separated by electrophoresis using polyacrylamide gel electrophoresis gels (4–12 %; LonzaIbérica S.A.U., Barcelona, Spain) and transferred to a polyscreen polyvinylidene difluoride membrane (Perkin Elmer, Waltham, MA, USA). After blocking with 5 % non-fat dried milk or 5 % bovine serum albumin, membranes were incubated with the respective primary antibodies—cleaved caspase3 (Asp175) antibody (#9661s) and p21 Waf1/Cip1 (12D1) antibody (#2947; Cell Signalling Technology)—overnight at 4 °C. The membranes were then incubated with the appropriate secondary horseradish peroxidase-conjugated IgG antibodies (GE Healthcare Europe GmbH, Barcelona, Spain) at a 1:3,000 dilution for 1 h at room temperature. Blots were visualized with ECL reagent (Pierce Biotechnology, Rockford, IL, USA) using a LAS4000 Lumi-Imager (Fuji Photo Film, Valhalla, NY, USA). *β*-Actin—ACTB antibody (A-2066; Sigma-Aldrich Química, S.L., Madrid, Spain)—served as the loading control. Protein spots were quantitated with Image J software (http://rsb.info.nih.gov/ij/index.html).

### 3-(4,5-Dimethylthiazol-2-yl)-2,5-diphenyltetrazolium bromide (MTT) assay

The viability of ECs was assessed by the colorimetric MTT reduction assay. Cells were plated in 96-well plates at a concentration of 4 × 10^3^cells/well and cultured in NG, HG or HM conditions for 21 days, with or without teneligliptin/sitagliptin, changing the media each 48 h. After treatments, cell medium was removed and replaced with media containing 0.5 mg/ml MTT and then incubated for 4–6 h at 37 °C in a humidified atmosphere with 5 % CO_2_. Formazan crystals formed were solubilized with 0.04 N HCl in isopropanol. The absorbance was measured at 570 nm, with a reference filter at 650 nm. The results are presented as mean ± SEM and represent the percentage of control.

### 5-Bromodeoxyuridine (BrdU) incorporation assay

This method was used to determine the cellular proliferation with a direct non-radioactive measurement of DNA synthesis, based on the incorporation of the pyridine analogous 5 bromo-2′-deoxyuridine (BrDu) instead of thymidine into the DNA of proliferating cells. BrdU incorporation was assayed using the Cell Proliferation enzyme-linked immunosorbent assay colorimetric assay (Roche, Mannheim, Germany) according to the manufacturer’s instructions. Briefly, HUVECs were grown at 2.5 × 10^3^ at 96-well plate and cultured in NG, HG or HM conditions for 21 days, with or without teneligliptin/sitagliptin, changing the media each 48 h. After this period, cells were labeled overnight with BrdU, and then they were fixed and washed. Anti-BrdU-POD working solution and substrate solution were added, and BrdU incorporation was quantified by measuring the absorbance at 370 nM in a microplate reader (Synergy HT, BioTek Instruments, Inc., Winooski, Vermont, USA).

### ROS measurement

The fluorescent probe, 2′,7′-Dichlorofluorescein diacetate (H_2_DCFDA), was used to measure the intracellular production of ROS. In all, 5 × 10^3^ HUVECs were grown on clear flat bottom treated 96-well plates during 21 days under NG, HG or HM conditions. At the end of the experiment, cells were treated with the indicated drugs, and the reactions were stopped staining cells with 20 μM H_2_DCFDA for 30 min at 37 °C. The fluorescence intensity of H_2_DCFDA was kinetically measured at an excitation and emission wavelength of 485 nm and 530 nm for H_2_DCFDA using a fluorescent microplate reader (Synergy HT, BioTek Instruments, Inc., Winooski, Vermont, USA.

### Statistical analysis

Numerical data were expressed as mean ± SD and they were analyzed using one-way ANOVA to compare the means of all the groups. The Bonferroni correction for multiple comparisons was used to determine which pairs of means were different. Differences were considered significant at *p* < 0.05.

## Results

### Chronic teneligliptin treatment at doses between 0.1 and 3.0 µmol/L does not reduce cell viability of HUVECs

We firstly performed an MTT analysis with the aim to examine whether HUVECs viability was affected due to teneligliptin addition at different doses. HUVECs were maintained for 21 days under different conditions: NG, HG and HM. During the exposure, teneligliptin was added chronically to the medium at 0.1, 1.0 or 3.0 μmol/L. We used as control the DPP-4i sitagliptin at the concentration of 0.5 μmol/L [13].

**Fig. 1:**
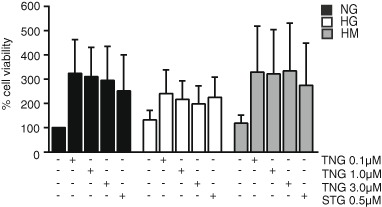
Teneligliptin effects on HUVECs viability after exposure to high**-**glucose conditions. HUVEC cells were incubated during 21 days under three different glucose conditions: NG (normoglycemia-5 mmol/L), HG (hyperglycemia-25 mmol/L) and NM (metabolic high-glucose memory-alternatively 5 and 25 mmol/L). Different doses of teneligliptin (0.1, 1.0 and 3.0 µmol/L) and sitagliptin (0.5 µmol/L) were used. Cell viability (%) was examined by an MTT assay. Bars represent mean ± SEM for three independent experiments

We did not observe a reduction in cell viability, but rather an increase, despite of non-significant values due to high variability between experiments at lower doses of the DPP-4i. Moreover, no differences between the lower teneligliptin doses were identified (Fig. 1).

### Chronic teneligliptin treatment decreases HG-stress markers in HUVEC cells

**Fig. 2:**
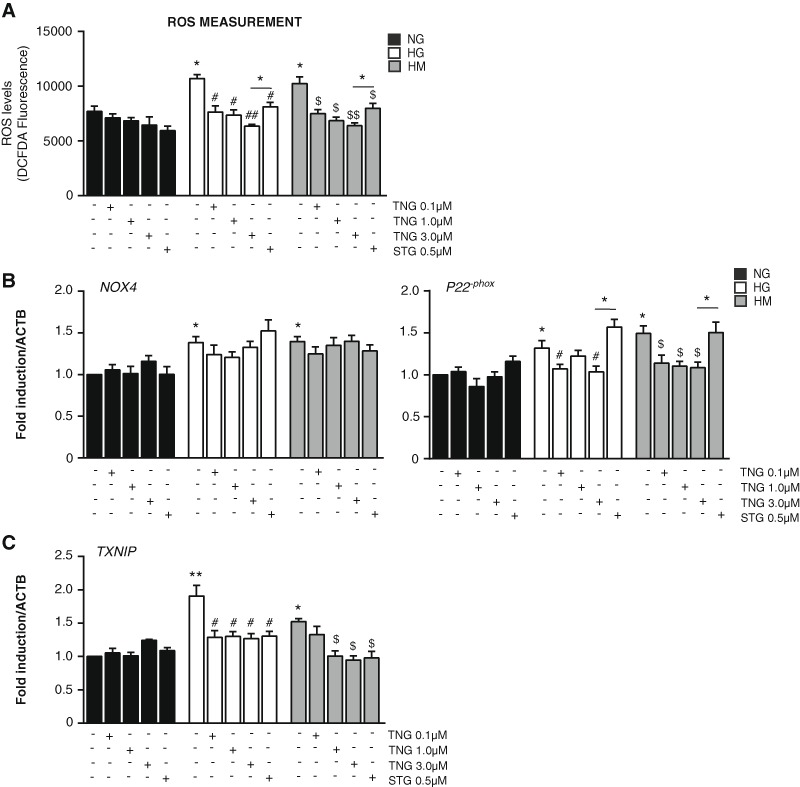
Teneligliptin effects on HG stress markers: ROS, NAD(P)H oxidase subunits and TXNIP in HUVECs cultured under HG/HM conditions. HUVECs were maintained under NG, HG or HM conditions during 21 days. During the exposure, teneligliptin was added chronically to the medium at 0.1, 1.0 or 3.0 μmol/L. We used as control the DPP-4 inhibitor sitagliptin at the concentration of 0.5 μmol/L. **a** ROS production was stained by 20 μM H_2_DCFDA for 30 min, and its oxidation product (DCF) fluorescence indicated ROS formation. **b**,**c** Total cellular RNA was isolated from HUVECs and mRNAs encoding for *NOX4*, *P22* ^−*phox*^ and *TXNIP* genes were assessed by qRT-PCR and expressed relative to *ACTB*. *p < 0.05 and **p < 0.01 vs. NG. #*p* < 0.05 vs. HG. $*p* < 0.05 vs. HM. Bars represent mean ± SEM for six independent experiments

We wondered to examine if a reduction of hyperglycemic stress after DPP-4i treatment existed. Three different markers of HG stress were then measured (Fig. 2): (1) the ROS; (2) the subunits *Nox4* and *P22* ^*−phox*^ of the enzyme NAD(P)H Oxidase, shown to be a source of ROS in the endothelium of diabetic patients [15]; and (3) the thioredoxin interacting protein (Txnip), which has been demonstrated to be increased under hyperglycemia and ROS overproduction [16, 17].

With the aim to assess the effects of teneligliptin on ROS production, HUVECs were exposed to HG, with or without glucose normalization, and then loaded with the cell-permeable ROS-sensitive fluorescent dye H_2_DCFDA. As expected, exposure to HG, either sustained or after normalization, resulted in a significant induction of fluorescence compared with cells exposed to NG. Teneligliptin treatment decreased ROS generation levels in those cells maintained under HG and HM conditions in a dose-dependent manner (Fig. 2a). A reduction in ROS production was also reached with sitagliptin, however, a significant difference was observed when HUVECs treated with teneligliptin 3.0 μmol/L were compared with cells treated with sitagliptin (Fig. 2a). Noteworthy, the efficacy of teneligliptin at 0.1 μmol/L was similar to 0.5 μmol/L sitagliptin, suggesting a more potent antioxidant property of teneliglitin.

ROS are constantly produced by all cellular types, and one of the involved reactions is through NAD(P)H oxidase (NOX), the predominant source of ROS in the vasculature [16]. We examined gene expression levels of the essential subunit *NOX4* and the cytosolic regulatory component *P22* ^−*phox*^ [18]. The expression of both *NOX4* and *P22* ^−*phox*^ was induced similarly under HG and HM conditions (Fig. 2b). Teneligliptin decreased only the messenger RNA (mRNA) of the regulatory subunit *P22* ^−*phox*^ that was reduced in a significant statistical manner. In contrast, sitagliptin did not exert any effect in the expression of *NOX4* and *P22* ^−*phox*^ (Fig. 2b).

TXNIP belongs to the thioredoxin (TRX) pathway, inhibiting TRX activity in a redox-dependent manner, by binding its reduced form but not the oxidized one [16]. TRX also has a redox-regulatory activity, playing a role in controlling signalling pathways that are involved in ROS production in endothelium [19]. In our cellular model, *TXNIP* expression is increased in HG and HM conditions, confirming the HG-induced endothelial damage also in HUVECs, being more pronounced in the HG state. Teneligliptin, as well as sitagliptin, treatments were able to counteract *TXNIP* upregulation, so proving the positive actions of both DPP-4i on HUVECs exposed under hyperglycemic conditions without normalization (Fig. 2c).

### Chronic teneligliptin treatment increases heme oxygenase-1 (HMOX1) gene expression in HUVEC cells incubated under hyperglycemia

**Fig. 3:**
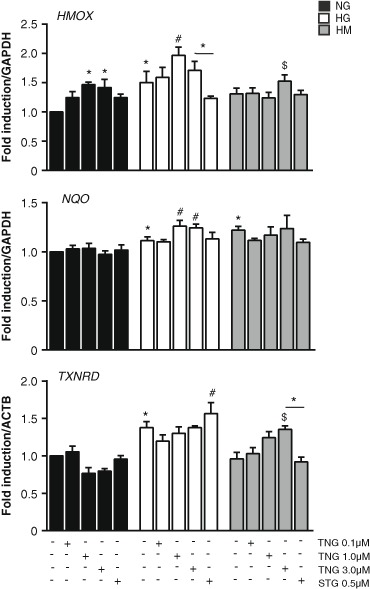
Teneligliptin effects on the antioxidant gene expression in HUVECs cultured under HG/HM conditions**.** Total cellular RNA was isolated from HUVECs after 21 days in culture in NG, HG or NM. Cells were treated with different doses of teneligliptin (0.1, 1.0 and 3.0 µmol/L) or sitagliptin (0.5 µmol/L). mRNA encoding for the indicated genes was assessed by qRT-PCR and expressed relative to *GAPDH* or *ACTB*. *p < 0.05 vs. NG. #*p* < 0.05 vs. HG. $*p* < 0.05 vs. HM. Bars represent mean ± SEM of six independent experiments

One of the potential targets of ROS, induced by NAD(P)H oxidase, is the transcription factor Nrf2, which is considered the master regulator of intracellular antioxidant response [16]. We analyzed gene expression levels of: HMOX, NAD(P)H dehydrogenase quinone-1 (NQO) and thioredoxin reductase (TXNRD), all described as Nrf2 target genes [16] and we observed that they responded in a different manner to HG and HM exposure, with or without DPP-4i treatment (Fig. 3).

In normoglycemia, teneligliptin upregulated *HMOX* gene expression in a dose-dependent manner, however, sitagliptin failed to do so. Sustained hyperglycemia exposure induced the expression of *HMOX* and *NQO* mRNA, indicating a possible activation of Nrf2 and the initiation of the antioxidant cascade. Teneligliptin enhances the antioxidant response in the higher doses (1.0 and 3.0 µmol/L), while sitagliptin had no effect, even a non-significant reduction in *HMOX*. The scenario was different in the HM condition. Only *NQO* was increased due to hyperglycemia, however, neither teneligliptin nor sitagliptin exerted any effect. Contrary, *HMOX* expression was reduced with the glucose normalization but teneligliptin increases its expression, only at its highest dose (Fig. 3).

Nrf2 controls not only Hmox-1 and Nqo-1 which are related to ROS detoxification process, but also other antioxidants pathways [16]. One of them is the TRX pathway, which is regulated not only by TXNIP, as described before, but also by TXNRD [16]. A different expression pattern was observed for the antioxidant *TXNRD*, a lower expression trend was observed by higher doses of teneligliptin in NG. TXNRD transcript levels were also regulated differently in chronic hyperglycemia and metabolic memory conditions: sustained hyperglycemia, but not metabolic memory state, induced *TXNRD* gene expression. Contrary to what expected, chronic teneligliptin treatment not only did not enhance such increase, but downregulated its gene expression for the HG state. Only sitagliptin was able to upregulate *TXNRD* in a significant manner in this condition. Different regulation was observed in HM, while TXNRD expression was not affected by the glycemic treatment, there was a dose-dependent upregulation of gene expression levels of *TXNRD* when HUVECs were treated with teneligliptin, reaching significance at its highest dose. No effect was observed after sitagliptin addition (Fig. 3).

### Chronic teneligliptin treatment improves proliferative capacities of HUVEC cells

In order to assess the pro-survival capacities of teneligliptin in HUVEC cells exposed to sustained HG conditions, gene expression levels of several markers of cell proliferation and apoptosis were analyzed: Cdkn1a/p21 (hereafter p21), Cdkn1b/p27 (hereafter p27), p53, Bcl-2, Bax and caspase 3.

P21, a member of the cyclin-dependent kinase inhibitors family is a downstream target of p53, it is upregulated following the persistent generation of ROS and functions as one of the major determinants of the cellular response to stress [20]. P21 also has pro-survival functions in response to oxidative stress by inhibiting the activation of caspase 3, caspase 9 and other pro-apoptotic factors, preventing cell death [21]. Some of the mediators of p21 protection system against hyperglycemia-induced cell death belong to the Bcl-2 family and, among them, the anti-apoptotic Bcl-2 and the pro-apoptotic Bax genes [22].

**Fig. 4:**
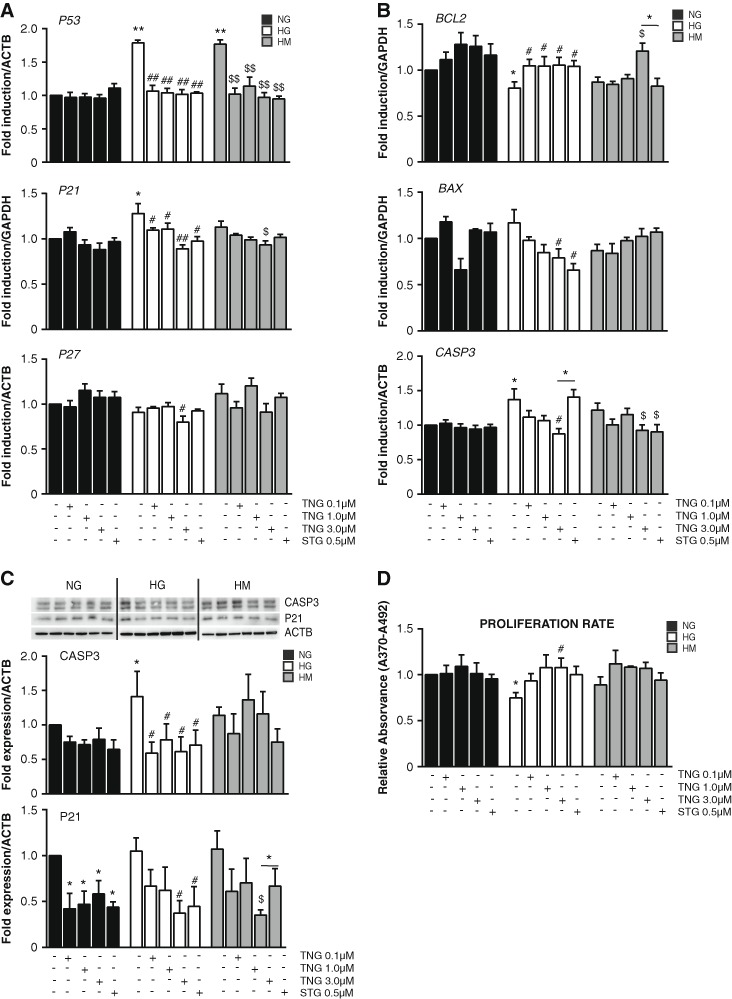
Teneligliptin effects on proliferation in HUVECs cultured under HG/HM conditions. HUVECs were maintained under NG, HG or HM conditions during 21 days. During the exposure, teneligliptin was added chronically to the medium at 0.1, 1.0 or 3.0 μmol/L. We used as control the DPP-4 inhibitor sitagliptin at the concentration of 0.5 μmol/L. **a**,**b** Total cellular RNA was isolated from HUVECs and mRNAs encoding for *NOX4* and *P22* ^−*phox*^ genes were assessed by qRT-PCR and expressed relative to *GADPH* or *ACTB*. **c** Protein expression of CASPASE 3 and P21 was assessed by western blot. The panels show a representative image of different independent experiments. Densitometric values were normalized to ACTB and represented relative to the control cells (NG), normalized to 1. **d** HUVECs proliferation was examined by measuring BrdU incorporation. *p < 0.05 and **p < 0.001 vs. NG. #*p* < 0.05 and ##*p* < 0.001 vs. HG. $*p* < 0.05 and $$*p* < 0.001 vs. HM. Bars represent mean ± SEM for six independent experiments

As expected, *p53* was increased when HUVECs were exposed to HG and HM conditions. Teneligliptin treatment added under these conditions produced a decrease in *p53* transcript levels. Sitagliptin had similar effects under the mentioned conditions (Fig. 4a). Regarding p21 gene expression, it was increased under HG conditions, and, contrary to what we expected, it was recovered under HM state. Teneligliptin was able to reduce its mRNA levels under all three conditions: NG, HG and HM. Such decrease was in a dose-dependent manner, and become statistically significant for the HG and HM states when teneligliptin was used at the highest concentration of 3.0 μmol/L. Sitagliptin also downregulated gene expression levels of *p21*, although not so important as teneligliptin 3.0 μmol/L did (Fig. 4a). P21 protein analysis confirmed the above mentioned results. Hyperglycemia increased P21 protein levels in a trend manner, and teneligliptin reduces its expression at all doses, even in normoglycemic condition. Similar observations can be described with sitagliptin addition (Fig. 4c).

Conversely, *P27* expression levels were not affected by hyperglycemia exposure, however, teneligliptin added at its highest dose in HG significantly decreased *p27* mRNA levels (Fig. 4a).

Regarding the apoptotic pathway, gene expression levels of the anti-apoptotic *BCL2* were increased by teneligliptin under NG conditions, and the pro-apoptotic gene *BAX* was decreased only when HUVECs were treated with teneligliptin 1.0 μmol/L. When ECs were exposed to HG conditions, gene expression levels of *BCL2* decreased and, in accordance, *CASP3* was upregulated, similarly to what is happening in HM (Fig. 4b). Teneligliptin was able to increase gene expression levels of *BCL2* in HG at similar levels with all doses, and in parallel it decreased *BAX* and *CASP3* expression in a dose-dependent manner, reaching significance only at the highest dose. Active CASP3 protein levels were also reduced by all doses of teneligliptin in HG (Fig. 4c). Not many changes in any of the aforementioned genes could be observed in HM, despite a reduction in *BCL2,* and concordantly in *CASP3* mRNA, when teneligliptin was added at 3.0 μmol/L (Fig. 4b). Sitagliptin had similar or lower effects to those observed for teneligliptin in these tested proliferation markers.

To confirm these results, next step was to assess if teneligliptin had effects on the proliferation rates of HUVECs. To this aim, HUVECs were cultured during 21 days under above mentioned experimental conditions and a BrdU analysis was performed. As it was expected, a significant decrease in the proliferation ratio in HUVECs cultured under chronic HG was observed, which was attenuated in the HM condition (Fig. 4d). Teneligliptin treatment significantly dose-dependently increased the proliferation rates of cells maintained in HG, as well as sitagliptin did. A similar, but non-significant, profile of proliferation stimulation was observed in HUVECs treated with teneligliptin at HM, but not with sitagliptin (Fig. 4d).

### Chronic teneligliptin treatment improves endoplasmic reticulum (ER) function in HUVEC cells exposed to HG

The pathological role of ER stress in the pathogenesis of diabetes and hyperglycemia is increasingly recognized [23]. Diabetes induces ER stress in many organs such as pancreas and liver [24], heart [25], nervous system [26], adipose tissue and kidney [27].

**Fig. 5:**
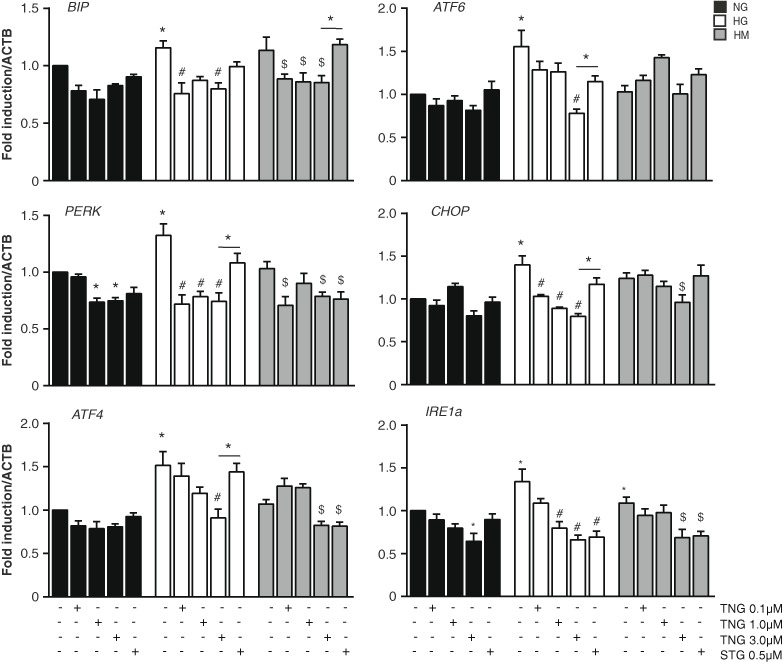
Teneligliptin effects on ER function in HUVECs cultured under HG/HM conditions. HUVECs were maintained under NG, HG or HM conditions during 21 days. During the exposure, teneligliptin was added chronically to the medium at 0.1, 1.0 or 3.0 μmol/L. We used as control the DPP-4 inhibitor sitagliptin at the concentration of 0.5 μmol/L. Total cellular RNA was isolated from HUVECs and mRNAs encoding for the different ER stress markers were assessed by qRT-PCR and expressed relative to *ACTB*. *p < 0.05 and vs. NG. #*p* < 0.5 vs. HG. $*p* < 0.5 vs. HM. Bars represent mean ± SEM for six independent experiments

The ability of cells to respond to perturbations in ER function is critical for their survival, and chronic or unresolved ER stress can lead to apoptosis [16, 28]. We assessed the expression of ER stress response-related transcripts under NG, HG and HM conditions in HUVEC cells. As we previously reported [29, 30], HUVEC cells exposed to HG and generally to the metabolic high-glucose memory, the expression of some ER stress markers and unfolded protein response (UPR) was increased. In HG, we observed an increase in the gene expression of the chaperon *BIP* and this result was in accordance with an increased UPR activity. Teneligliptin was able to decrease gene expression levels of *BIP* at all doses both in HG and HM, even sitagliptin had any significant effect (Fig. 5).

We also checked the gene expression of several downstream markers of the three different signalling cascades described for UPR: Perk, Ire1a and Atf6. Regarding Perk pathway, we assessed the mRNA expression of *PERK*, *ATF4* and *CHOP*. Teneligliptin added in NG condition did not affect mRNA levels of *ATF4* or *CHOP*. However, teneligliptin treatment was able to reduce *PERK* gene expression levels when it was added at 1.0 and 3.0 μmol/L doses. Furthermore, as expected, all these transcripts were increased both under HG and HM conditions, even if reaching significance in the HG condition, and this result was in accordance with the published data based on the demonstration of hyperglycemia-induced damage in ER homeostasis [24, 25, 31].

HUVECs exposed to HG or HM treated with teneligliptin respond differently for each gene. In HG, downregulation due to teneligliptin administration was in a dose-dependent manner for the genes *ATF4* and *CHOP*, while for *PERK* we observed a drastic decrease just for the low doses. Sitagliptin had no significant effect in any of them. In HM, teneligliptin was efficient in reducing *ATF4* and *CHOP* expression only at its highest dose, while *PERK* was reduced with all. Whereas, sitagliptin had no effect on *CHOP* expression in HM, it reduced *PERK* and *ATF4* at similar levels than teneliglitptin did (Fig. 5).

Teneligliptin reduced *IRE1a* gene expression in a dose-dependent manner, being significant only at high dose of teneligliptin. HG and HM conditions also increased *IRE1a*, and teneligliptin treatment was able to decrease mRNA levels in both unhealthy conditions, like sitagliptin did (Fig. 5). Finally, teneligliptin added in NG did not affect *ATF6* mRNA levels. As in generally observed for the other transcripts, HG exposure induced an increase in gene expression levels of *ATF6*, but no effect was further observed after glucose normalization. Teneligliptin treatment was able to reduce *ATF6* mRNA levels at its highest dose in HG, while sitagliptin was not (Fig. 5).

## Discussion and conclusion

DPP-4 inhibitors are potent drugs used to treat patients with T2DM [32]. Inhibition of DPP-4 increases levels of biologically intact incretins, improving glucose metabolism through the upregulation of insulin secretion and the suppression of glucagon release [7]. Several preclinical studies have demonstrated that incretin therapies have cardioprotective actions, reducing atherosclerosis, vascular injury and ED. Incretin-based large-scale clinical trials in T2DM patients with GLP-1RA demonstrate that GLP-1 receptor agonist, liraglutide, reduced the rates of cardiovascular events and death compared to the placebo in patients with type 2 diabetes who were at high-risk for cardiovascular events (LEADER trial), although lixisenatide, which is the short-acting form, did not show any cardiovascular benefit in patients with diabetes and a recent acute coronary syndrome (ELIXA trial) [33]. On the other hand, the outcome studies (SAVOR-TIMI53, EXAMINE and TECOS) demonstrated no cardiovascular benefit in patients treated with DPP-4i, although cardiovascular safety was not compromised [34–36]. In contrast, the newly published results of the SPEAD-A study, in which T2DM patients without CVD history treated with alogliptin, showed a reduced progression of the carotid intima-media thickness in treated patients [37]. A recent study evidences the discrepancy between preclinical and clinical data using these drugs, like DPP-4i, presumably due to the usage of different experimental models that give rise to diverse and sometimes non-correlated effects [32]. So, these results highlighted the need to gain further insights on the molecular mechanisms of DPP-4i, before they can induce changes in current clinical practice. Moreover, the synthesis of new agents, as well as longer follow up for complications development could lead to different results. It may be that new agents, which more powerful ancillary actions may show different results.

Teneligliptin has different structure and pharmacodynamics characteristics from other gliptins, features that could confer properties diverse from or additive to other DPP-4i. First, it is a potent, selective and long-lasting inhibitor of DPP-4 and exhibits strong inhibitory activity via its J-shaped structure and ‘anchor lock domain’. The second important feature is that teneligliptin has high tissue distribution [38], which may be because of its hydrophobic property. Since DPP-4 is expressed in various tissues, including vasculature, this might explain teneligliptin pleiotropic effect [10], as well as its putative protective role in CVD.

In addition to its DPP-4 inhibitory activity, teneligliptin has radical scavenging properties and showed to normalize the increased levels of 8-hydroxy-2-deoxyguanosine in urine, kidney and aorta of diabetic rats [39]. Another study [40] demonstrated that long-term treatment with teneligliptin significantly improves endothelial function in an in vivo model of spontaneously hypertensive stroke prone rats that exhibit insulin resistance and glucose intolerance.

Recently, it has been shown that 24-week teneligliptin treatment improves endothelial function by reactive hyperemia index, by reducing the oxidative stress markers reactive oxygen metabolites (ROMs), measured by the d-ROMS test, 8-hydroxy-2′-deoxyguanosine (8-OHdG) and the urinary liver-type fatty acid-binding protein in patients with T2DM and chronic kidney disease, compared to patients who had received sitagliptin for at least 12 months prior to switching to teneligliptin. On the other hand, glucose-lowering effect was not significantly different between the two groups [41]. This study suggests that the antioxidative and renoprotective effects of teneligliptin might be stronger than those observed with other DPP-4 inhibitors, particularly sitagliptin, which might be independent of blood glucose-lowering effect.

The main findings of the present study are:

(i) Teneligliptin has antioxidant properties under NG in HUVECs, reducing ROS levels and initiating the transcriptional cascade of the antioxidant genes;
(ii) HG and HM conditions cause a decrease in proliferation in HUVEC cells, and teneligliptin has the ability to recover these capacities;
(iii) ER function is impaired due to HG and HM conditions; teneligliptin can ameliorate it and improve ER homeostasis.

As far as we know, this is the first time that a direct action of a DPP-4i on ROS production is described in an endothelial cellular model. Other studies described the capacity of DPP-4i, sitagliptin or alogliptin, to counteract ROS production, in other experimental model: such as the ApoE^−/−^ mice [41, 42]. Here, we demonstrated that teneligliptin at all used doses, and in a dose-response manner, is able to restore ROS production levels impaired by either HG or HM detrimental conditions. It is noteworthy that teneligliptin has a better effect than sitagliptin counteracting ROS production, even at low doses, meaning a powerful action of teneliglitptin improving the redox state of ECs.

The novelty of this study is to show how teneligliptin initiates the antioxidant transcriptional cascade at mRNA level in an endothelial cellular in vitro model, promoting the expression of Nrf2 target genes such as *HMOX*, *NQO* or *TXNRD*, and normalizing the levels of *TXNIP* which is upregulated by HG. Strikingly, differently from sitagliptin, teneligliptin is able to induce the antioxidant Nrf-2-related transcriptional cascade, even under HG and HM conditions.

The effect of hyperglycemia-induced ER dysfunction has become a focus of increasing research. The ER is a dynamic tubular network that participates, among its various actions, in the maturation and the proper folding of proteins [14]. ER dysfunction has been proposed as one of the causes of *β*-cell loss of function [40], and damage in other tissues, such as the adipose tissue [41, 42], the liver [43] and the cardiovascular system [44].

Our cellular model confirms that HUVECs cultured under HG and HM conditions have an increased gene expression of several ER markers. Novelty, we showed that the DPP-4i teneligliptin lowers the expression of these ER stress markers in HUVECs cultured in NG. Moreover, it counteracts negative effects produced when ECs are exposed to HG with or without normalization. When the UPR mechanism fails leading to the continuous accumulation of unfolded proteins, and the cell is not able to re-establish ER homeostasis, the apoptotic cascade is initiated through the activation/inactivation of Bcl-2 family members [16]. As expected, HG lowers cell proliferation rate and increases apoptosis. Teneligliptin added at NG does not induce any change in proliferation, however, when it is added under HG or HM states, it is able to counteract the apoptotic phenotype induced by hyperglycemia restoring proliferation capacities of HUVECs. Although HUVECs are widely used in the vasculature studies, they are distinct from ECs relevant to adult T2DM in terms of vascular bed-umbilical vein vs. arteries and arterioles or higher proliferation rate compared to adult ECs such as human aortic endothelial cells (HAECs). Therefore, further studies need to be addressed to confirm these results in other cellular and specific in vivo models.

In conclusion, this study suggests that long-term treatment with the DPP-4 inhibitor teneligliptin can induce antioxidant-response increasing the expression of NRF2 targets, with consequent oxidative stress amelioration and decrease in ROS production. Moreover, teneligliptin has a protective role reducing HG-induced ER stress, promoting cellular recovery, measured as attenuated apoptosis and increased proliferation rate.

## Abbreviations

Apoptosis signalling kinase-1: ASK-1
*β*-Actin: ACTB
Bovine serum albumin: BSA
5-Bromodeoxyuridine: BrdU
Cardiovascular diseases: CVD
Cyclin-dependent kinase inhibitor-1a/b: Cdkn-1a/b
2′,7′-Dichlorofluorescein diacetate: H_2_DCFDA
3-(4,5-Dimethylthiazol-2-yl)-2,5-diphenyltetrazolium bromide: MTT
Dipeptidyl peptidase-4: DDP-4
DPP-4 inhibitors: DPP-4i
Endothelial cells: ECs
Endothelial dysfunction: ED
Endoplasmic reticulum: ER
Fetal bovine serum: FBS
Gastric inhibitory polypeptide: GIP
Gentamicin/amphotericin: GA
Glucagon-like peptide-1: GLP-1
Heme oxygenase-1: HMOX-1
High glucose: HG
High-metabolic memory: HM
Human aortic endothelial cells: HAECs.
Human epidermal growth factor: hEGF
Human recombinant fibroblast growth factor-beta: hFGF-b
Human umbilical vein endothelial cells: HUVECs
NAD(P)H dehydrogenase quinone-1: NQO-1
NAD(P)H oxidase: NOX
Nicotine amide adenine dinucleotide phosphate: NADPH
Non-fat dried milk: NFDM
Normal glucose: NG
Nuclear factor (erythroid-derived 2)-like 2: NRF2
Reactive oxygen species: ROS
Real-time polymerase chain reaction: qRT-PCR
Thioredoxin: TRX
Thioredoxin interacting protein: TXNIP
Thioredoxin reductase: TXNRD
Type 2 diabetes mellitus: T2DM
Unfolded protein response: UPR
